# Changes in lipids composition and metabolism in colorectal cancer: a review

**DOI:** 10.1186/s12944-019-0977-8

**Published:** 2019-01-26

**Authors:** Alicja Pakiet, Jarosław Kobiela, Piotr Stepnowski, Tomasz Sledzinski, Adriana Mika

**Affiliations:** 1 Department of Environmental Analysis, Faculty of Chemistry, University of Gdansk, Gdansk, Poland; 2 Department of Pharmaceutical Biochemistry, Faculty of Pharmacy, Medical University of Gdansk, Dębinki 1, 80-211 Gdansk, Poland; 3 Department of General, Endocrine and Transplant Surgery, Faculty of Medicine, Medical University of Gdansk, Gdansk, Poland

**Keywords:** Colorectal cancer, Polar lipids, Oxylipins, Fatty acids, Lipidomics, Metabolism

## Abstract

Altered metabolism of lipids is currently considered a hallmark characteristic of many malignancies, including colorectal cancer (CRC). Lipids are a large group of metabolites that differ in terms of their fatty acid composition. This review summarizes recent evidence, documenting many alterations in the content and composition of fatty acids, polar lipids, oxylipins and triacylglycerols in CRC patients’ sera, tumor tissues and adipose tissue. Some of altered lipid molecules may be potential biomarkers of CRC risk, development and progression. Owing to a significant role of many lipids in cancer cell metabolism, some of lipid metabolism pathways may also constitute specific targets for anti-CRC therapy.

## Introduction

Finding a disease, the course of which is not related to lipid alterations can be challenging. Lipids raise a growing interest as potential biomarkers in many clinical conditions. This highlights the importance of lipidomic studies in understanding, diagnosing and treating numerous human pathologies, among them cancer; the use of lipidomics could create an opportunity to design targeted therapies, prognostic or screening biomarkers [1]. In everyday clinical practice, lipid status is estimated based on serum concentrations of total cholesterol (TC), high density lipoprotein (HDL), low density lipoprotein (LDL) and triacylglycerols (TGs). While only a limited information can be obtained from the analysis of those lipid fractions, other currently available techniques, e.g. mass spectrometry, may provide a detailed insight into the structure and function of some specific lipid species. In this review paper, we discuss lipid alterations associated with colorectal cancer (CRC), with special emphasis on fatty acids (FAs) and their potential therapeutic and diagnostic applications in patients with this malignancy.

Most cancers found in the colon or rectum are adenocarcinomas arising from pathological lesions in the epithelial cells of colorectal mucosa [2]. Vast majority of CRCs are thought to evolve from conventional adenomas through as a result of several dozens of mutations; this process is referred to as the adenoma-to-carcinoma sequence [3]. Most CRCs are sporadic malignancies and are not associated with inherited mutations in established cancer-related genes [4]. However, about 20–30% CRC may be associated with inherited mutations [5]. A progressive accumulation of multiple genetic mutations contributes to transition from normal mucosa to benign adenoma, severe dysplasia, and eventually, a frank carcinoma. It is estimated that approximately 15% of sporadic colon cancers are a consequence of malfunction in mismatch repair genes, whereas other 80–85% are associated with mutations in adenomatous polyposis coli (APC) gene. Furthermore, colon cancer may develop as a consequence of inflammatory bowel disease, on a different, yet uncharacterized pathway. Malignant transformation requires further genetic alterations [6]. Less than 50% of colon cancers harbor mutated KRAS, a protein that is involved in intracellular signal transduction [7, 8]. Approximately 50% of colonic lesions with high-grade dysplasia and about 75% of frank cancers may carry p53 mutations [6, 7]. A neoplastic disease cannot be effectively managed without the understanding of distinctive characteristics of cancer cells that contribute to tumor development. One of them is enhanced proliferation [9]. Two main genetic defects found in CRC, KRAS and p53 mutations, are both associated with enhanced proliferation [10, 11]. Intensively proliferating cancer cells display some unique metabolic patterns due to which they may obtain enough energy for new biomass synthesis. Cancer cells have a unique ability to generate energy in a nutrient-deficient environment. Since the preference of cancer cells for glycolysis rather than oxidative phosphorylations (OXPHOs) when oxygen is not limited has been demonstrated by Otto Warburg [12], the aberrant glucose metabolism became one of the hallmarks of cancer. However there has been a paradigm shift towards so called reversed Warburg effect, since research showed that each cancer has its unique metabolic features, and some may synthesize ATP by means of OXPHOs [13]. A recent evidence suggests that CRC cells rely on the reversed Warburg effect [14, 15], which opened new perspectives for the identification of new molecular therapeutic targets, among them FA oxidation [16]. Another frequently observed characteristic of cancer cells is their dependence on exogenous glutamine. Many oncogenic mutations seem to affect glutamine metabolism, which may open new therapeutic perspectives [17, 18]. Aside from the switch in glucose and glutamine metabolism, lipids may also play a role in the adaptation of cancer cells. It is well known that cancer cells show alterations of lipid metabolism. This may lead to structural changes in their membranes, disruption of energy homeostasis, cell signaling, gene expression and protein distribution, affecting a number of cell functions, such as apoptosis, autophagy, necrosis, proliferation, differentiation, growth, drug and chemotherapy resistance [19–22]. The role of lipids and their metabolism in cancer development and spread raises a growing interest of researchers, as shown in previous reviews [23–25]. The lipid metabolic pathways that have been affected in CRC cells include synthesis, desaturation, elongation and mitochondrial oxidation of the FAs. CRC belongs to the three leading causes of mortality in both male and female cancer patients [26, 27]. Non-invasive tests for CRC, such as guaiac-based fecal occult blood test (gFOBT), as well as more sensitive, fecal immunochemical test (FIT) and stool DNA test, are usually conducted on stool samples [28, 29]. Colonoscopy is an invasive screening method, considered a gold standard for the detection of colorectal neoplasms. Other screening instruments include flexible sigmoidoscopy and newer techniques, such as colon capsule endoscopy and magnetic resonance colonography [30]. However, all these techniques are invasive, and hence, both patients and researchers await easy to determine and accurate blood-derived biomarkers. Typically, biological material used for research purposes includes biopsy specimens of colorectal mucosa, surgical specimens of colonic lesions, blood serum or plasma and red blood cells (RBCs). However, this is blood which is particularly useful from the perspective of biomarker research, as it can be obtained more easily and less invasively than other biological materials.

### The lipidome changes in colorectal cancer patients

Lipidomics, a distinct branch of metabolome studies, provides information about the role of lipid dysregulation in various pathological conditions, such as metabolic syndrome [31], obesity [32], non-alcoholic fatty liver disease [33], diabetes [34] and cardiovascular diseases [35]. A growing number of studies analyzed the relation between lipids and various malignancies: breast cancer [36, 37], prostate cancer [38, 39], ovarian cancer [40, 41], hepatocellular carcinoma [42], lung cancer [43], pancreatic cancer [44] or bladder cancer [45]. Understanding the link between the disease and lipidome not only provides a better insight into its pathogenesis but is also vital for the development of novel biomarkers and therapeutic strategies.

**Fig. 1:**
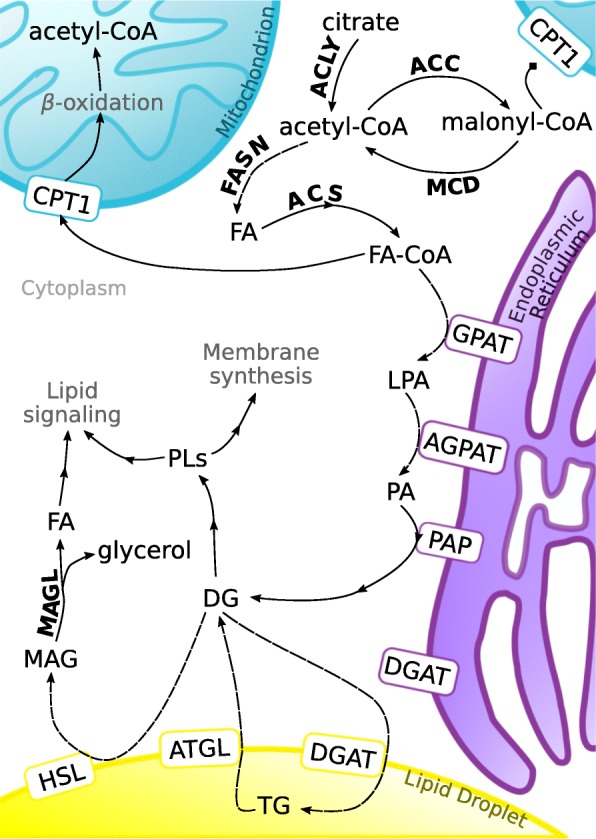
Overview of endogenous metabolism of fatty acids at the cellular level. Modified from Currie et al. (2013) [24]. DG: diacylglycerol, FA: fatty acid, LPA: lysophosphatidic acid, MAG: monoacylglycerol, MUFA-CoA: monounsaturated fatty acid-coenzyme A, TG: triacylglycerol, ACC: acetyl-CoA carboxylase, ACS: acetyl-CoA synthetase, ACLY: ATP citrate lyase, FASN: fatty acid synthase, MAGL: monoacylglycerol lipase, MCD: malonyl-CoA decarboxylase, AGPAT: 1-acylglycerol-3-phosphate-O-acyltransferase, ATGL: adipose triglyceride lipase, CPT1: carnitine palmitoyltransferase I, DGAT: diacylglycerol O-acyltransferase, GPAT: glycerol-3-phosphate acyltransferase, HSL: hormone sensitive lipase, PAP: phosphatidate phosphatase

Lipids are a diverse group of compounds belonging to various species. LIPID MAPS [46] classified them into eight groups based on the presence of ketoacyl and isoprene groups: FAs, glycerolipids, glycerophospholipids, sphingolipids, sterol lipids, prenol lipids, saccharolipids and polyketides [47]. The properties of various lipids and their biological functions change depending on the head-group [48]. The main structural component of each lipid group are fatty acids. FAs are structural components of complex lipids and play a wide range of roles in human body. FAs building phospholipids (PHLs) constitute the main fraction of lipid bilayer. Aside from serving as structural components, FAs, in form of glycerol esters (i.e. TGs), serve also as an energy reservoir in adipose tissue. Upon release from TGs by adipose tissue lipases, they are delivered to various organs as circulating free fatty acids (FFAs) [49]. FAs can be classified into three groups: saturated FAs (SFAs), without double bonds in acyl chain, monounsaturated FAs (MUFAs), with one double bond, and polyunsaturated FAs (PUFAs), with more than one double bond in acyl chain. Furthermore, they can be classified based on the number of carbon atoms, as short-chain FAs (SCFAs), with up to 6 carbons, medium-chain FAs (MCFAs), with 6 to 12 carbons, and long-chain FAs (LCFAa), with more than 12 carbons [50]. FAs can be both endo- and exogenous. De novo synthesis of palmitic acid from acetyl-CoA (acetyl-coenzyme A) is catalyzed by fatty acid synthase (FASN) [20] and other enzymes, among them those responsible for converting citrate to oxaloacetate and acetyl-CoA, and for carboxylation of acetyl-CoA. To enter bioactive pool, FAs needs to be activated, as FA-CoA. SFAs are converted into MUFAs by stearoyl-CoA desaturase 1 (SCD-1, Δ-9-desaturase) [24, 25] and their chains are elongated by elongases (ELOVLs) [51]. The activated FAs may serve as substrates for the synthesis of TGs and PHLs or are transported to mitochondria whereby they undergo oxidation. The synthesis of endogenous FAs and their metabolism are presented schematically in Fig. 1. Some FAs cannot be synthetized by human cells and must be provided with the diet. Both 18:3 n-3 FAs found in some plant oils (flaxseed, rapeseed, canola), walnuts and leafy greens, and 18:2 n-6 FAs contained in meat, poultry, cereal products and oil, are essential fatty acids [52, 53]. They act as precursors for the synthesis of longer n-3 and n-6 PUFAs, competing for the same enzymes [54]. Long-chain n-3 PUFAs can be also provided with fish products, the global consumption of which is generally too low [55]. The activity of FAs and complex lipids they build is determined by their structure. The length of acyl chain and the degree of its saturation determine various functions of FAs, e.g. the rigidity of plasma membranes and biological effects in humans. SCFAs synthesized by gastrointestinal microbiota, especially butyrate, are the primary energetic substrate for colonocytes, promote colonic health and have anti-inflammatory properties [50]. The degree of unsaturation determines the susceptibility of unsaturated FAs to oxidation; PUFAs are generally more prone to oxidation due to the presence of multiple double bonds [56]. Also position of the double bond within the PUFA molecule is vital from the functional perspective, since n-6 PUFA metabolites are generally proinflammatory whereas n-3 PUFAs act as anti-inflammatory compounds. Therefore, the role of lipids in various pathological conditions should be analyzed not only at the group level, but also at the species level. Concentration of circulating non-esterified fatty acids (NEFAs), also referred to as FFAs, may be a predictor of metabolic status in various conditions associated with obesity [57]. Available evidence suggests that obesity may be associated with increased risk of CRC [58, 59]. Elevated level of FFAs in serum may be a marker of oxidative stress [60], enhanced lipotoxicity [61] or hypertriglyceridemia.

### Alterations of saturated fatty acids in CRC

#### Dietary and endogenous saturated fatty acids

The tumor development is associated with enhanced lipogenesis [24, 62–64]. De novo lipogenesis was shown to be associated with enhanced saturation of membrane lipids in colorectal cancer cell line, HCT116. SFAs, abundant due to increased activity of FASN, are incorporated into membrane PHLs, making the cells less susceptible to free radicals and penetration of therapeutics [65]. Increased activity of FASN is also associated with β-oxidation of endogenous lipids and promotion of cellular respiration [20]. These processes are induced by mammalian target of rapamycin kinase (mTOR kinase) signaling pathway, which activates the synthesis of proteins being responsible for growth, division or angiogenesis during tumorigenesis [66, 67] and metastasis [68]. Wang et al. demonstrated that FASN knockdown results in downregulation of cancer invasion and spread in cell lines [69]. While cancer cells generally show preference for endogenous FAs, some malignancies may also require provision of exogenous fatty acids [70].

According to one hypothesis, an increase in the incidence of CRC in eastern populations may result from a change in dietary preferences in favor of SFA-rich western style diet [71]. A primary dietary source of SFAs are animal products. High dietary intake of SFAs has been implicated in obesity-associated gene expression profile and metabolic syndrome [72, 73] and was shown to impair white adipose tissue function [74] and to induce insulin resistance [75]. The supplementation of monocytic cell lines with SFAs may activate nuclear factor κB, upregulate cyclooxygenase-2 and toll-like receptors 2 and 4 (TLR2 and TLR4) [76]; the TLRs are known to play a role in carcinogenesis associated with inflammation [77]. Dietary intake of SFAs has also an impact on serum lipoproteins. Substitution of cis-PUFAs and MUFAs with an equivalent amount of energy from dietary SFAs contributed to an increase in serum levels of total cholesterol, HDL-cholesterol, LDL-cholesterol and triglycerides, although this effect was statistically significant only in the case of PUFA replacement [78]. The results of previous studies analyzing a link between dyslipidemia and CRC are inconclusive [79–82]. However, dietary modifications alone may not be enough to explain the etiopathogenesis of a given condition; for example, while a short-term change in diet showed only a minimal correlation with genes in the pathway of an inflammatory marker, prostaglandin E2 (PGE2), an increase in colonic SFAs stimulated a rise in PGE2 concentration [83]. Although a dietary intake of SFAs (estimated with a diet questionnaire) correlated positively with rectal cancer risk, in contrast to fatty acids of plasma PHL, it showed no significant association with the overall risk of CRC [84].

#### Enhanced elongation of saturated fatty acids in CRC

According to Kondo, CRC patients had significantly lower serum levels of long-chain FAs, 14:0, 15:0 and 18:0, and presented with significantly higher serum levels of very long-chain FAs (VLCFAs), 24:0, 25:0, 26:0, 28:0 and 30:0, than healthy controls [85]. However, Zhang observed more than a 50% increase in 18:0 content in cancerous tissue, but without concomitant changes in 14:0 and 16:0 levels [86]. The Singapore Chinese Health Study showed that while rectal cancer patients and healthy subjects did not differ significantly in terms of 16:0 and 18:0 plasma PHL levels, the 16:0 content correlated inversely with colon cancer risk [87]. In turn, Mendelian randomization analysis pointed to a significant relation between 18:0 content and CRC risk [88]. Some studies demonstrated that CRC was associated with a significant increase in serum level of VLCFAs [85, 89]. Our previous study showed that cancerous tissue contained more 22-, 24- and 26-carbon SFAs and MUFAs than normal colonic tissue [22]. Moreover, we found 26:0 cerotic acid exclusively in the sera of CRC patients, and hence, proposed it as a serum biomarker of this malignancy [22]. However, Kondo observed only a 1.33-fold increase in serum 26:0 in CRC patients [85]. The lack of 16:0 accumulation implies that the products of FASN may serve as substrates for other enzymes. The activity of various elongating enzymes ELOVLs may be cancer type-specific, e.g. ELVOL7 is involved in prostate cancer [90], ELOVL2 in breast cancer [91] and ELOVL1 and ELOVL 6 in triple negative breast cancer [36]. Enhanced activity of elongases in CRC tissue, assessed based on elongation index values (18:0/16:0 and 22–26:0/20:0 ratios) and higher ELOVL1 and ELOVL6 mRNA levels, contributed to an increase in saturated and monounsaturated VLCFA content in tumor tissue, and probably was also a reason behind the elevated serum concentrations of VLCFAs. Plausibly, the latter may serve as a biomarker of CRC [22].

### Endogenous and exogenous monounsaturated fatty acids

Oleic acid (18:1 n-9, OA) is one of the most abundant FAs in human tissues and the most abundant MUFA [22, 86, 92]. It is the main dietary MUFA provided with both animal products and plant oils, especially with olive oil. The latter is an essential component of Mediterranean diet [93], which has been implied to protect against cancer [94]. However, the exact mechanism through which OA would interfere with CRC is still not fully understood. Both oleic acid and palmitic acid trigger non-canonical autophagic response in human cancer cells, but through different mechanisms (beclin-1-independent autophagy that requires intact Golgi apparatus or via the activity of 5’AMP-activated protein kinase (AMPK), protein kinase R (PKR) and c-Jun N-terminal protein kinase 1 (JNK1)) [95]. The role of autophagy in cancer progression is ambiguous; while it can suppress cancer development at its early stages, preventing accumulation of mutated cells or aggregation of reactive oxygen species (ROS), it can also boost the resilience of cancer cells via supply of extra energy whenever nutrients are sparse [96]. Moreover, oleic acid was shown to decrease mRNA levels for some FA transporters and receptors and to reduce lipid droplet content in colonic adenocarcinoma cells [97]. The level of oleic acid was demonstrated to be significantly reduced in CRC tumor tissue, which was associated with a shift from stage B to stage C in Dukes classification [86].

MUFAs can be also synthesized in vivo by SCD-1, an enzyme expressed in all major tissues, as well as by SCD-5, found in the pancreas and brain [98]. Available evidence points to a link between various cancer types and SCD-1 expression [99–102]. Overexpression of SCD-1 and other enzymes, namely ATP-binding cassette sub-family A member (ABCA1), long chain acyl-CoA synthetase (ACSL1) and 1-acyl-sn-glycerol-3-phosphate acyltransferase alpha (AGPAT1), was associated with increased risk of recurrence and worse outcomes in stage II colon cancer [103]. However, the upregulation of SCD-1 was not reflected by an increase in serum and tissue levels of 18:1, plausibly because of the incorporation of this FA into complex lipids or its utilization as a substrate for other enzymes involved in lipid metabolism [86, 87]. The relation between cancer mortality and activity of SCD-1, estimated on the basis of serum cholesteryl ester ratio of 16:1 n-7 and 16:0, and presence of a single nucleotide polymorphism in its gene, suggests that endogenous synthesis of MUFAs may exert an effect on cancer outcome [104]. As a result, SCD-1 became a target for anticancer therapy [105, 106]. ACSL/SCD-1 pathway can regulate the invasiveness of cancer cells and serve as a predictor of survival. Further, silencing of SCD-1 with siRNAs was shown to activate apoptosis in HCT116 cells [107].

### n-3 and n-6 polyunsaturated fatty acids

PUFAs are represented by two families, n-3 and n-6, which exert opposite effects on inflammation. n-3 PUFAs are known to exhibit anti-inflammatory properties. Inflammation is one of the hallmarks of cancer. Patients with inflammatory bowel disease have increased risk of CRC [108, 109]. n-3 PUFAs can attenuate inflammation via multiple mechanisms, inter alia acting via their oxidized derivatives [110, 111]. Long chain n-3 PUFAs, specifically 20:5 n-3 (eicosapentaenoic acid, EPA) and 22:6 n-3 (docosahexaenoic acid, DHA) found in oily fish, were shown to interfere in vitro with the kappa-light-chain-enhancer of activated B cells (NF-κB) signaling system, downregulating nuclear NF-κB p65 component and NF-κB inhibitor (IκBα) and upregulating cytoplasmic NF-κB p50 in a time- and dose-dependent manner [112]. The upregulation of NF-κB was observed in some cell lines, including human CRC cells [111]. While n-3 PUFAs were shown to exert an anti-inflammatory effect, published data about the link between consumption of fish or supplementation with fish oil and the risk of CRC are inconclusive. While some observational studies demonstrated that dietary provision of n-3 PUFAs from those sources was associated with a decrease in CRC risk [113] and lower mortality from that malignancy [114], others did not find enough evidence to support this link [115]. Although a recent meta-analysis of nine studies demonstrated an overall improvement in the levels of inflammatory markers, IL-6, albumin and CRP/albumin ratio, it also documented difficulties in obtaining comparable data about the anti-inflammatory effects of DHA or EPA supplementation; the authors proposed that at least some of the results might have been influenced by inconsistencies in supplementation protocols [116]. Moreover, it must be stressed that n-3 PUFA supplements may contain some proportion of n-6 PUFAs and SFAs, which also might interfere with their beneficial effects [117]. Finally, the supplements in which a major n-3 PUFA is ALA may offer limited advantages due to low impact on DHA and EPA levels resulting from limited conversion rates on n-3 PUFA pathway [118].

Another mechanism through which n-3 may exert a beneficial biological effect and prevent carcinogenesis, is disruption of lipid rafts associated with their low affinity for cholesterol and saturated chains and resultant lesser rigidity of formed structures [119, 120]. Proteins embedded in lipid rafts were shown to be involved in cell signaling, proliferation, adhesion and apoptosis [121, 122]. Turk et al. reported that DHA but not EPA or 20:4 n-6 (arachidonic acid, AA) enhanced phosphorylation of epidermal growth factor receptor (EGFR) and reduced downstream signaling in young adult mouse colonic (YAMC) cells and in a murine model [123]. Enhanced phosphorylation of EGFR after supplementation with n-3 PUFAs was also observed in breast cancer cell lines [124]. Those findings are worth emphasizing as EGFR is also known to be overexpressed in most CRCs [125].

n-6 PUFAs are abundant in plant oils [126]. Most FAs from the n-6 PUFA family, especially AA and its oxidized products, show proinflammatory properties [127], and thus, may act as tumor promoters. However, the evidence from observational studies analyzing the role of AA in CRC risk is inconclusive [128]. High dietary n-6/n-3 PUFA ratio may be an important risk factor of other epithelial malignancies, such as aggressive prostate cancer [129], breast cancer [37] or invasive lung cancer [43]. Zhang et al. demonstrated that n-6/n-3 PUFAs ratio in cancerous tissue was significantly higher than in adjacent normal tissue [86], and this observation was later confirmed in another study [130]. However, caution has to be applied when studying effects of n-3 and n-6 PUFA metabolites. Relying simply on n-6/n-3 PUFA ratio may be a source of bias, since some n-6 derived oxidation products may in fact have anti-inflammatory properties [131].

Humans do not have the ability to synthesize linoleic acid (LA, 18:2 n-6) and α-linolenic acid (ALA, 18:3 n-3) de novo due to the lack of Δ-12-desaturase and Δ-15-desaturase [132]. Previous studies demonstrated that CRC patients can be distinguished from healthy controls and individuals with colorectal polyps based on their serum levels of LA and ALA [133, 134]. In another study, CRC patients presented with nearly 50% lower serum concentrations of 18:3 n-6 (γ-linolenic acid, GLA) than healthy controls [85]. Additionally, GLA was proposed as a biomarker for CRC risk because its altered concentrations could be observed as early as at the adenoma stage, but without an evident decreasing or increasing tendency across stages I to IV [85]. Another study showed that the level of 18:2 n-6 in cancerous tissue was significantly higher than in adjacent normal tissue, and differed depending on Dukes stage [86]. However, an opposite relationship was found in another larger study including more than twice as many CRC patients, in which 18:2 n-6 concentration in cancerous tissue was significantly lower than in normal tissue and did not correlate with TNM stage [130]. Certain proportion of dietary 18:2 n-6 and 18:3 n-3 are converted to long-chain PUFAs by elongation and desaturation. Specifically, 18:3 n-3 is a substrate for EPA and DHA, whereas 18:2 n-6 is converted to AA through combined action of elongases, Δ-5 and Δ-6 desaturases [52]. However, the product of 18:2 n-6 elongation can be also converted by Δ-5-desaturase to a unique n-6 PUFA, dihomo-γ-linolenic acid (DGLA) 20:3 n-6, which exerts an opposite biological effect to AA [126, 135]. According to Butler et al., the plasma indices of n-6 PUFA desaturation pathway correlated positively with increased colon cancer risk [87].

The growing popularity of dietary supplements containing conjugated linoleic acids (CLA) and their widely reported beneficial effects observed in animals, inter alia anticancer activity [136], stimulated research on the link between CLAs and CRC. The term ‘CLA’ refers to the group of LA isomers, both cis and trans, with conjugated double bonds. The main natural dietary source of CLAs are ruminant-derived dairy products that contain primarily *cis*-9 and *trans-*11 isomers [137]. In turn, commercially available supplements are racemates of cis-9, trans-11, trans-10 and cis-12 CLAs. Two mechanisms of action of CLAs have been proposed. First, CLAs may reduce the level of harmful COX-2 metabolites [138], and second, they may act as ligands for peroxisome proliferator-activated receptors (PPARs) [137, 139]. Some studies demonstrated beneficial effects of CLAs in cell lines [140] and murine models [137] and a decrease in tumor invasiveness and improvement of inflammatory status were observed in CLA-supplemented rectal cancer patients [141, 142]. However, CLAs should be used with caution in cancer patients, as the study in healthy volunteers demonstrated that their administration may cause loss of appetite, which would pose a risk of cachexia in persons with malignancies [143].

### Products of lipid oxidation

Link between oxidative stress, chronic inflammation and an array of chronic disorders have been studied extensively in cardiovascular diseases [144], diabetes mellitus [145], rheumatoid arthritis [146] and cancer [147–149]. Oxidative stress damages various molecular species, including proteins, nucleic acids and lipids. Oxidized lipid products may be formed either during a non-specific peroxidation facilitated by oxidative stress, as lipid peroxidation products (LPPs), or be generated in enzymatic reactions catalyzed by cyclooxygenases (COX), lipoxygenases (LOX) and cytochromes p450 (CYP450), as oxylipins. Due to presence of multiple double bonds, PUFAs are most susceptible to oxidation; while n-3 PUFA-derived oxylipins generally produce favorable biological effects, the products of n-6 PUFA oxidation usually have proinflammatory properties.

#### Products of oxidative stress

Carcinogenesis is associated with oxidative stress [150, 151]. The breakdown of PUFAs and PUFA-containing complex lipids starts from the formation of lipoperoxyl radical, a product of ROS-mediated damage to a susceptible double bond. A reaction between the lipoperoxyl radical and lipid molecules results in formation of lipid radicals and lipid hydroxyperoxides, which are further degraded to secondary products [56]. The markers of oxidative stress are isoprostanes (isoPs), containing primarily F-type prostane rings derived from AA, and DHA-derived neuroprostanes [152]. Among non-enzymatically formed arachidonic acid metabolites series 2 isoprostanes, CRC patients presented with lower serum levels of 8-iso-PGF2α and elevated serum concentrations of 2,3-dinor-8-iso-PGF2α [153]. The end-products of ROS-mediated lipid breakdown are 4-hydroxynonenal (HNE) and malondialdehyde (MDA), both found at elevated concentrations in CRC tissues [154]. Both MDA and 4-HNE are established mutagenics in humans [155, 156]. However, 4-HNE may also exert an anticancerogenic effect, as it was shown to inhibit the activity of telomerase in Caco-2 and HT-29 cell lines [157].

#### Enzymatically formed pro- and anti-inflammatory oxylipins

**Fig. 2:**
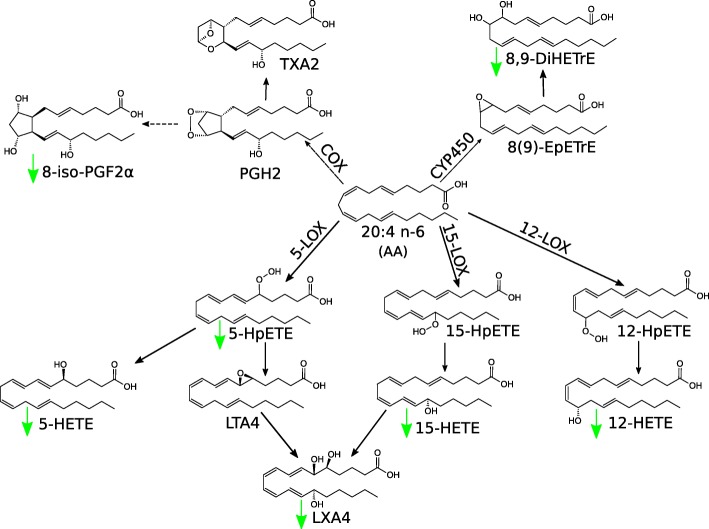
Synthesis of eicosanoids in colorectal cancer cells. Green arrows indicate direction of change of the level of compounds in serum of CRC patients [153]. COX: cyclooxygenase, CYP450: cytochrome P450, DiHETrE: dihydroxyeicosatrienoic acid, EpETrE: epoxyeicosatrienoic acid, HETE: hydroxyeicosatetraenoic acid, HpETE: hydroperoxyeicosatetraenoic acid, LOX: lipoxygenase, LT: leukotriene, LX: lipoxin, PG: prostaglandin, TX: thromboxane

Oxidized lipid species can be also generated in enzymatic reactions catalyzed by COXs, LOXs and CYP450. The process begins with the release of FA from membrane phospholipid. The reaction is catalyzed by an enzyme from cytosolic phospholipase A_2_ family (cPLA_2_) [158]. However, also adipose TG lipase (ATGL) has been implicated as an enzyme involved in the release of substrate for oxylipin production from mast cell membranes [159]. Baseline concentration of oxylipins seems to be modulated by dietary intake of PUFAs [160]. To this date, the most extensively studied oxylipins have been AA derivatives, referred to as eicosanoids, since cPLA_2_α shows a preference for AA release [158] enabling downstream enzymes to synthesize more than 50 AA derivatives [161]. However, some eicosanoids, namely resolvins, protectins and maresins, may be also synthesized from n-3 PUFAs [32]. Chemical structures of some representative eicosanoids synthesized from AA on various enzymatic pathways are shown in Fig. 2. Oxidation of AA on COX-2 pathway results in generation of series 2 oxylipins: prostaglandins and thromboxanes [162]. Previous studies demonstrated that concentration of prostaglandin PGE_2_ correlated positively with cancer stem cell (CSC) markers in human colorectal tumor samples; furthermore, PGE_2_ was shown to promote CSC expansion in a murine model [163]. However, the available evidence in this matter is inconclusive, since according to Zhang et al., serum concentration of PGE_2_ and its product, 20-hydroxy-PGE_2_ in CRC patients were significantly lower than in healthy controls [153]. The group of LOX-derived AA oxylipins includes hydroxyeicosatetraenoic acids (HETEs), also synthesized on CYP450 pathway, and leukotrienes. 12S-HETE was shown to promote the invasiveness of colorectal adenocarcinoma cells via activation of myosin regulatory light chain 2 (MLC2), Rho/Rho-associated coiled-coil containing protein kinase (Rho/ROCK) and Ca^2+^ signaling [164]. Leukotrienes are inflammatory mediators synthesized from AA and EPA on 5-LOX (5-lipooxygenase) pathway. LTB4 (leukotriene B4) is a well-established pro-inflammatory compound; its proinflammatory activity is inter alia associated with its ability to promote formation of reactive oxygen species [165]. 12-keto-LBT4, an inactive product of LBT4 conversion via LTB4–12-hydroxydehydrogease/15-oxo-prostaglandin-13-reductase (LTB4DH/15oPGR), was shown to be considerably downregulated in CRC patients sera, and thus, has been proposed as a potential biomarker of this malignancy [153]. Moreover, 15S-HETE concentration is significantly lower in serum of CRC patients that was not associated with Duke’s stage, which suggests drop in its levels early in cancer development [166]. 15S-HETE is a metabolite of AA known for its anti-inflammatory properties. As an antagonist of cancer promoting 20-HETE, 19-HETE may prevent proliferation of cancer cells. Indeed, one study showed that CRC patients presented with lower serum concentrations of 19-HETE and lower values of 19-HETE/20-HETE ratio [153]. Also, some specialized pro-resolving mediators (SPMs) were analyzed for their association with adenoma occurrence risk. However, blood levels of neither lipoxin A4 (a product of AA) nor resolvin D1 (a derivative of DHA) were accurate enough to identify patients with a past history of adenoma [167]. According to Ritchie, an inverse correlation between TNM stage and serum concentrations of some hydroxylated, polyunsaturated ultra-long-chain fatty acids (hPULCFAs) were found in CRC patients participating in an untargeted biomarker discovery study [168]. hPULCFAs resemble some derivatives of FAs, namely lipoxins, resolvins and protectins [168], but their exact structure and biological role are yet to be explained. Further studies demonstrated that a moiety with molecular mass of 446 (C_28_H_46_O_4_), referred to as GTA-446, may be a marker of CRC risk in healthy persons [168, 169] and is more sensitive than blood gFOBT [170]. However, other authors put into question the predictive value of GTA-446, and proposed that it could be rather used as a diagnostic marker [171].

### Polar lipids in blood and tissues of CRC patients

Polar lipids (PLs) are the most abundant lipids in cells and inner compartment membranes. Thus, the structure of PLs determines physical properties of membrane bilayer; a change in the degree of saturation of FAs that build PLs may affect membrane fluidity and consequently, also its permeability. Analysis of plasma PLs in CRC patients revealed altered profiles of FAs, namely an increase in total SFAs and a decrease in PUFA content [172]. Published evidence suggests that an increase in SFA fraction of plasma PLs may be associated with greater risk of CRC [84], colon cancer [87], and colon adenoma [173]. Incorporation of SFAs contributes to ER stress-induced apoptosis [174]. Additionally, also an increase in elaidic (trans-9 18:1) PL fraction correlated with adenoma presence [173].

Mass spectrometry-based imaging studies documented an increase in MUFA content, positive correlation with the levels of PC-32:1, PC-34:1 and PC-36:1 phosphatidylcholines (PCs) in cancerous tissue, and a downregulation of polyunsaturated FAs and polyunsaturated PLs, except from a 1.49-fold increase in phosphatidic acid PA-36:2 [175]. Another signature of CRC seems to be a significant upregulation of PC-16:0/16:1 [176], lysophosphatidylcholines LPC-16:0, LPC-18:1 and PC-16:0/18:1 [177]. The authors of one lipidomic study demonstrated considerable alterations of several complex plasma lipids in CRC patients, and based on the analysis of receiver operating characteristic (ROC) curve proposed phosphatidylglycerol PG-18:0/16:0, sphingomyelin SM-d18:1/24:1 (42:2), ceramide Cer-d18:1/26:4 (elevated), LPC-18:3, LPC-18:2, phosphorylethanolamines PE-18:2/18:1, PE-18:1/20:2 and SM-38:8 (decreased) as biomarkers of this malignancy [89]. The use of biomarker clusters may have greater discriminative power than single molecules. In one study, patients with early CRC were identified accurately based on their serum levels of palmitic amide, oleamide, hexadecanedioic acid, 12-hydroxystearic acid, 20:3 n-3, 14:0, lysophosphatidic acid LPA-16:0, LPA-18:0 and LPC-16:0, with the area under the ROC curve equal 0.991, 0.981 sensitivity and 1.000 specificity [178]. Similar approach, with a panel of various metabolites, among them lipids, was also used to predict the recurrence and spread of CRC and survival in patients with this malignancy [179]. Also, the activity of enzymes involved in PL metabolism may be altered in cancer patients. Upregulation of choline kinase α (CHα) results in an increase in PC content, whereas the overexpression of lysophosphatidylcholine acyltransferases, LPCAT1 and LPCAT4, contributes to alterations of PL profiles [175, 180]. In one study, cancer tissue contained elevated levels of PA-36:2 and less PA-38:3, PA-40:5, PE-38:4, sphingomyelins SM-22:0 and SM-22:4 [175].

Sphingolipids (SPLs) are a group of complex lipids, containing a sphingoid base as a backbone, linked to fatty acid chain. SPLs can be either synthesized de novo from L-serine and palmitoyl-CoA in endoplasmic reticulum yielding membrane-bound dihydroceramide, or originate from the degradation of complex sphingolipids on the salvage pathway [181, 182]. CRC patients may present with elevated plasma levels of some glycosphingolipids containing glucose (Glu) or lactose (Lac), namely GluCer-42:3, GluCer-42:2, GluCer-36:4, GluCer-34:1, GluCer-33:2, LacCer-42:4, LacCer-40:1, LacCer-40:2, LacCer-40:4, LacCer-38:1 and LacCer-35:1 [89]. CRC tissues were shown to contain more Cer-16:0, Cer-24:0 and Cer-24:1, and less Cer-18:0 and Cer-22:0, as well as elevated mRNA levels for ceramidase synthases, CerS1, CerS2, CerS5 and CerS6 [183, 184].

Hartman et al. found that Cer present in HCT-116 colon cancer cell line originated primarily from de novo synthesis [185]. Overexpression of CerS4 leads to inhibition of cell proliferation and an increase in Cer-16:0 content. In turn, upregulation of CerS6 was shown to be associated with an increase in Cer-18:0 and Cer-20:0 levels. In CerS2-overexpressing cells, supplementation with nervonyl- or lignoceryl-CoA resulted in upregulation of very long chain-containing Cer species, Cer-24:0 and Cer-24:1, and enhanced proliferation. Further studies showed that the activity of CerS2 may partially depend on ELOVL1 expression [186]. Also an important role of a balance between long- and very long-chain FA-containing Cer was emphasized on the basis of the observation on diminished apoptosis in the case of CerS4/CerS6 and CerS2 co-expression [186]. Also Shen reported on elevated plasma levels of Cer that contained long-chain FAs (Cer-d18:1/26:4) in CRC patients [89].

Cer are proapoptotic molecules involved in stress-induced signaling pathways, among them, in JNK pathway [187, 188]. The inhibition of SCD-1 in human adenocarcinoma LOVO cells was shown to be associated with a significant decrease in proliferation rate and accumulation of saturated endoceramides, Cer-16:0 to Cer-24:0. The overproduction of Cer-18:0 to Cer-24:0 ceased upon supplementation with 18:1 n-9. Administration of SCD-1 inhibitor caused a delay in tumor growth in xenograft mice, which could be reversed after blockade of Cer biosynthesis. These findings imply that Cer may influence the SCD-1-mediated apoptosis due to a cross-talk between these two pathways [184].

Cer is a central molecule to sphingolipid metabolism. Following cleavage of FAs by ceramidase, the remaining sphingosine can be phosphorylated by sphingosine kinases (SphK1 or SphK2) to form sphingosine-1-phosphate (S1P) [189]. Some studies demonstrated that tumor tissues contain significantly more SphK1 than normal colonic mucosa [190, 191]. SphK1 expression knockdown in colorectal adenocarcinoma cell lines was associated with a decrease in tumor cell migration and invasiveness, probably due to interference with epithelial-mesenchymal transition (EMT) [191], a process observed during cancer progression and spread [192].

To summarize, published evidence suggests that CRC may be associated with alterations in PLs. Since PLs are important structural and functional molecules involved in cell growth and differentiation, their alterations may also play a role in carcinogenesis [193]. The evidence from mass spectrometry studies points to PLs as potential cancer biomarkers, but diagnostic and prognostic value of those molecules still needs to be verified in large clinical studies [194, 195].

### Association between blood and tumor tissue triacylglycerols and CRC

TGs are the primary depot of highly-concentrated metabolic energy released from adipose tissue in form of FAs and delivered with blood to target tissues. Dietary TGs are hydrolyzed in the intestine, re-esterified in the enterocytes, conjugated with cholesterol and proteins in form of chylomicrons, and eventually, released into the blood. Moreover, TGs may originate from endogenous synthesis in the liver and be released in the form of very-low-density-lipoproteins (VLDL). Elevated concentration of TGs observed during the course of dyslipidemia is an established risk factor of cardiovascular disease [196]. Published data about the link between blood TGs and CRC risk are inconclusive. According to some authors, elevated serum or plasma levels of total TGs were associated with increased risk of colorectal adenoma [197], colonic adenoma risk [198]; the relationship seemed to be stronger for the colon than for the rectum, and in men than in women [199, 200]. However, other studies did not demonstrate a link between serum/plasma TGs and CRC risk [79, 201–203]. A meta-analysis of published studies dealing with the problem in question suffered from a considerable heterogeneity of source data, since the study populations differed markedly in their CRC risk profiles, probably due to variations in environmental factors [204]. Furthermore, little is known on specific FAs forming TGs. Serum TGs of Min mice (a mouse model of colorectal cancer) showed greater level of hydroxyperoxidation and contained elevated concentrations of TG species with 18:2 n-6 chains, especially during polyp formation [205]. Rapid evaporative ionization mass spectrometry (REIMS) imaging demonstrated that CRC tissues contained significantly less TG 54:0 than benign adenomas; however, concentration of this TG in CRC was still significantly higher than in normal tissues [206]. In another study, rectal cancer patients showed a significant increase in serum TG 56:6, 52:2 and 52:1, but it must be stressed that the study group was relatively small [89]. The authors of most studies analyzing TG levels in blood and tissues of CRC patients reported their overall concentrations but did not provide a detailed information about the content of specific FAs.

### Specific fatty acids changes in adipose tissue of CRC patients

Although available data on FAs esterified in TGs are generally limited, some studies provided an insight into this lipid group, based on the analysis of adipose tissue. The latter is the main reservoir of TGs, capable of releasing them into bloodstream, and thus, it may influence the lipid profiles of various tissues. Many studies documented a relationship between obesity and colorectal cancer risk [58, 59]. Abdominal fat deposits, which can be expressed as the waist-to-hip ratio, seem to be a predominant “measure” of colorectal adenoma risk in men and women [207]. Moreover, as outlined recently in the review articles published by Himbert [208] and Masoodi [209]; also multifaceted interactions between adipose microenvironment and tumor, especially those mediated by proinflammatory factors, raise a growing interest of researchers. Thus, adipose tissue is no longer considered a merely energy reservoir, but also a source of various signaling molecules, adipokines [210], and FAs with proinflammatory properties that can modulate immune cells [211] or activating autophagy [212]. Furthermore, adipose tissue is no longer analyzed as a single entity, but as two distinct compartments, visceral adipose tissue (VAT) and subcutaneous adipose tissue (SAT). Furthermore, studies of SAT sometimes consider additional heterogenic nature of this tissue, with two distinct layers, deep and superficial one, that differ in terms of various parameters, e.g. the intensity of lipolysis [213–216]. Surprisingly, however, only few previous studies analyzed a link between CRC occurrence or progression and the content of some specific FAs in adipose tissue, showing some significant changes of their levels [92, 213, 217, 218].

The authors of one study published in 1988 found no significant intergroup differences in the content of seven major FAs determined by means of GLC-FID in SAT and RBCs from 49 CRC patients and 49 sex- and age-matched controls [218]. Also another case-control study conducted by Giuliani et al. [92] showed no significant differences in total SFAs or MUFAs content between SAT and VAT for both controls and patient. Total SFA content in VAT and total MUFA content in SAT turned out to be higher in CRC patients than in the controls (*p* < 0.001). Among specific PUFAs, CRC patients presented with higher levels of visceral 18:3 n-3 whereas lower 18:4 n-3 than the controls. Furthermore, the study showed that in CRC patients, the level of n-6 PUFA, 18:2 n-6, was significantly higher in SAT than in VAT. Finally, the total content of n-6 PUFAs (LA + GLA + DGLA + AA) in SAT was shown to be higher in healthy controls than in CRC patients.

A somehow different approach was presented by Cottet et al. [217], who analyzed subcutaneous adipose tissue FAs based on the estimated activity of the enzymes involved in their metabolism. Therein the putative marker for ELVOL5 + Δ-6-desaturase activities estimated by 20:3 n-6 to 18:2 n-6 ratio as well as ELVOL2/5 activity (ratio of 22:4 n-6 to 20:4 n-6 and 22:5 n-3 to 20:5 n-3) were positively associated with CRC risk. No such association with CRC risk was observed on the basis of 18:1 n-9 to 16:1 n-9 ratio.

One limitation of adipose tissue studies is the method of sample preparation, which has already been shown to influence FA concentration [219]. Furthermore, adipose tissue collection is an invasive procedure, and hence, is unlikely to be applicable to large-scale studies.

## Conclusions

Despite a decrease in mortality, CRC still remains a serious public health burden [26]. A growing number of CRCs are diagnosed in patients younger than 50 years [220, 221]. The reason for this alarming tendency is yet to be elucidated, but it may be a consequence of greater exposure to environmental factors, lesser physical activity and unfavorable dietary changes. Analysis of lipid metabolism in cancer patients may provide a better insight into metabolic disturbances that contribute to carcinogenesis. The fact that cancer cells require lipids to proliferate [20], may open new therapeutic perspectives: perhaps some specific pathways involved in the synthesis and storage of fatty acids might be targeted to prevent cancer development [24]. Furthermore, some metabolites of fatty acids are important signaling molecules involved in the maintenance of proinflammatory and anti-inflammatory equilibrium. Probably these are proinflammatory factors which constitute a link between obesity and CRC [208]. Moreover, obesity is associated with lipidome changes [32] that may predispose to the development of some related conditions, among them cancer. Alterations of FAs, their metabolites and lipid species containing FA chains can be observed in tumor microenvironment as well (Table 1). Some of those alterations, such as accumulation of PC-16:0/16:1, may be considered as cancer biomarkers [176]. Lipid profile alterations, e.g. presence of cerotic acid [22] or a decrease in the concentration of hydroxylated, polyunsaturated ultra-long-chain fatty acids [169], can be also found in the sera of CRC patients, differentiating between early and advanced stages of this malignancy [178], or serving as a predictor of survival [179]. However, the development of clinically useful lipid biomarkers requires consistent research methodology, and previous studies were quite heterogenous in this matter. Another drawback of previous studies is limited sample size which may hinder generalization of their results onto the whole population of CRC patients. Nevertheless, understanding of lipid alterations associated with CRC may define new directions in the diagnosis and treatment of this malignancy.

**Table 1:** Colorectal cancer related changes of lipid species content in various biological samples

Research material	Lipid species	Lipid fraction	Direction of change	Reference
Cancer tissue	14:0	total lipids	↓	Mika et al. (2017) [22]
		total lipids	↑	Qiu et al. (2014) [179]
	16:0	total lipids	↓	Mika et al. (2017) [22]
			↓	Li et al. (2013) [178]
		ceramides	↑	Chen et al. (2015) [183]
			↑	Chen et al. (2016) [184]
		lysophosphatidylcholines	↑	Mirnezami et al. (2014) [177]
			↑	Li et al. (2013) [178]
		lysophosphatidic acid	↑	Li et al. (2013) [178]
	18:0	total lipids	↑	Mika et al. (2017) [22]
			↑	Zhang et al. (2013) [86]
		free fatty acids	↑	Chen et al. (2015) [183]
		total lipids	↓	Li et al. (2013) [178]
		ceramides	↓	Chen et al. (2015) [183]
			↓	Chen et al. (2016) [184]
		lysophosphatidylcholines	↑	Li et al. (2013) [178]
		lysophosphatidic acid	↑	Li et al. (2013) [178]
	20:0	total lipids	↑	Mika et al. (2017) [22]
		ceramides	↓	Chen et al. (2015) [183]
			↓	Chen et al. (2016) [184]
	22:0	total lipids	↑	Mika et al. (2017) [22]
		sphingomyelin	↓	Guo et al. (2014) [175]
	24:0	total lipids	↑	Mika et al. (2017) [22]
		free fatty acids	↑	Chen et al. (2015) [183]
		ceramides	↑	Chen et al. (2015) [183]
			↑	Chen et al. (2016) [184]
	26:0	total lipids	↑	Mika et al. (2017) [22]
	14:1	total lipids	↓	Mika et al. (2017) [22]
		free fatty acids	↑	Chen et al. (2015) [183]
	16:1 n-7	total lipids	↓	Mika et al. (2017) [22]
			↓	Zhang et al. (2013) [86]
		total lipids	↑	Qiu et al. (2014) [179]
	16:1 n-7	free fatty acids	↑	Chen et al. (2015) [183]
			↑	Guo et al. (2014) [175]
	18:1 n-9	total lipids	↓	Mika et al. (2017) [22]
			↓	Zhang et al. (2013) [86]
	18:1 n-9	free fatty acids	↑	Guo et al. (2014) [175]
			↑	Chen et al. (2015) [183]
	18:1 n-9	lysophosphatidylcholines	↑	Mirnezami et al. (2014) [177]
			↑	Li et al. (2013) [178]
	20:1	free fatty acids	↑	Guo et al. (2014) [175]
			↑	Chen et al. (2015) [183]
	22:1	total lipids	↑	Mika et al. (2017) [22]
	22:1	free fatty acids	↑	Chen et al. (2015) [183]
	24:1	total lipids	↑	Mika et al. (2017) [22]
	24:1	free fatty acids	↑	Chen et al. (2015) [183]
	24:1	ceramides	↑	Chen et al. (2015) [183]
			↑	Chen et al. (2016) [184]
	24:1	sphingomyelin	↓	Guo et al. (2014) [175]
	26:1	total lipids	↑	Mika et al. (2017) [22]
	18:2 n-6	total lipids	↑	Zhang et al. (2013) [86]
	18:2 n-6	total lipids	↓	Yang et al. (2015) [130]
	20:2 n-6	total lipids	↑	Zhang et al. (2013) [86]
	20:2 n-6	free fatty acids	↑	Chen et al. (2015) [183]
	20:4 n-6	total lipids	↑	Mika et al. (2017) [22]
			↑	Zhang et al. (2013) [86]
	20:4 n-6	free fatty acids	↓	Guo et al. (2014) [175]
	20:4 n-6	lysophosphatidylcholines	↓	Li et al. (2013) [178]
	20:3 n-6	total lipids	↑	Zhang et al. (2013) [86]
			↑	Yang et al. (2015) [130]
	22:4 n-6	free fatty acids	↓	Guo et al. (2014) [175]
	22:4 n-6	free fatty acids	↑	Chen et al. (2015) [183]
	20:5 n-3	total lipids	↑	Mika et al. (2017) [22]
			↑	Yang et al. (2015) [130]
	20:5 n-3	free fatty acids	↑	Chen et al. (2015) [183]
	20:5 n-3	total lipids	↓	Zhang et al. (2013) [86]
	20:5 n-3	free fatty acids	↓	Guo et al. (2014) [175]
	22:6 n-3	total lipids	↑	Mika et al. (2017) [22]
			↑	Yang et al. (2015) [130]
	22:6 n-3	free fatty acids	↑	Chen et al. (2015) [183]
	22:6 n-3	total lipids	↓	Zhang et al. (2013) [86]
	22:6 n-3	lysophosphatidylcholines	↓	Li et al. (2013) [178]
	malondialdehyde		↑	Skrzydlewska et al. (2005) [154]
	4-hydroxynonenal		↑	Skrzydlewska et al. (2005) [154]
	1,2-DG-36:3		↓	Alexander et al. (2017) [206]
	Cer-d18:0/H24:0		↑	Alexander et al. (2017) [206]
	Cer-t18:0/24:0(2OH)		↑	Alexander et al. (2017) [206]
	GlcCer-30:1		↑	Alexander et al. (2017) [206]
	PA-31:0		↑	Alexander et al. (2017) [206]
	PA-34:0		↑	Alexander et al. (2017) [206]
	PA-36:2		↑	Guo et al. (2014) [175]
	PA-38:3		↓	Guo et al. (2014) [175]
	PA-40:5		↓	Guo et al. (2014) [175]
	PC-16:0/16:1		↑	Kurabe et al. (2013) [176]
	PC-16:0/18:1		↑	Mirnezami et al. (2014) [177]
	PC-32:1		↑	Shen et al. (2017) [89]
	PC-34:1		↑	Guo et al. (2014) [175]
			↑	Li et al. (2013) [178]
	PC-36:1		↑	Guo et al. (2014) [175]
	PC-38:4		↓	Guo et al. (2014) [175]
	PC-38:6		↓	Guo et al. (2014) [175]
	PE-34:4		↑	Alexander et al. (2017) [206]
	PE-38:4		↓	Guo et al. (2014) [175]
	PG 38:4		↓	Alexander et al. (2017) [206]
	PG-36:1		↑	Alexander et al. (2017) [206]
	PI-38:4		↓	Guo et al. (2014) [175]
	PS-41:0		↑	Alexander et al. (2017) [206]
	PS-43:4		↓	Alexander et al. (2017) [206]
	PS-44:6		↑	Alexander et al. (2017) [206]
	PS-44:8		↑	Alexander et al. (2017) [206]
	SM-22:0		↓	Guo et al. (2014) [175]
	SM-24:1		↓	Guo et al. (2014) [175]
	TG-54:0		↓	Alexander et al. (2017) [206]
serum	14:0	total lipids	↓	Kondo et al.. (2011) [85]
		total lipids	↑	Mika et al.. (2017) [22]
	15:0	total lipids	↓	Kondo et al.. (2011) [85]
	18:0	total lipids	↓	Mika et al. (2017) [22]
			↓	Kondo et al. (2011) [85]
	22:0	total lipids	↑	Mika et al. (2017) [22]
	24:0	total lipids	↑	Kondo et al. (2011) [85]
	26:0	total lipids	↑	Mika et al. (2017) [22]
			↑	Kondo et al. (2011) [85]
	28:0	total lipids	↑	Kondo et al. (2011) [85]
	30:0	total lipids	↑	Kondo et al. (2011) [85]
	18:1 n-9	total lipids	↑	Mika et al. (2017) [22]
	26:1	total lipids	↑	Mika et al. (2017) [22]
	18:2 n-6	total lipids	↓	Zhu et al. (2014) [134]
	18:3 n-6	total lipids	↓	Kondo et al. (2011) [85]
	18:3 n-3	total lipids	↓	Mika et al. (2017) [22]
			↓	Zhu et al. (2014) [134]
	20:5 n-3	total lipids	↓	Mika et al. (2017) [22]
	9,10-DiHOME		↓	Zhang et al. (2017) [153]
	12,13-DiHOME		↓	Zhang et al. (2017) [153]
	9-HpODE		↓	Zhang et al. (2017) [153]
	9-HODE		↓	Zhang et al. (2017) [153]
	9-KODE		↓	Zhang et al. (2017) [153]
	13-HpODE		↓	Zhang et al. (2017) [153]
	13-HODE		↓	Zhang et al. (2017) [153]
	13-KODE		↓	Zhang et al. (2017) [153]
	19-HETE		↓	Zhang et al. (2017) [153]
	20-HETE		↓	Zhang et al. (2017) [153]
	12-keto-LTB4		↓	Zhang et al. (2017) [153]
	PGE2		↓	Zhang et al. (2017) [153]
	2-hydroxy-PGE2		↓	Zhang et al. (2017) [153]
	5-HpETE		↓	Zhang et al. (2017) [153]
	5-HETE		↓	Zhang et al. (2017) [153]
	LTD4		↑	Zhang et al. (2017) [153]
	LTE4		↑	Zhang et al. (2017) [153]
	14,15-LTE4		↑	Zhang et al. (2017) [153]
	12-keto-LTB4		↓	Zhang et al. (2017) [153]
	5S,6R-LXA4		↓	Zhang et al. (2017) [153]
	12-HETE		↓	Zhang et al. (2017) [153]
	15-HETE		↓	Zhang et al. (2017) [153]
			↓	Chen et al. (2003) [166]
	8-HETE		↓	Zhang et al. (2017) [153]
	14,15-DHET		↓	Zhang et al. (2017) [153]
	8,9-DHET		↓	Zhang et al. (2017) [153]
	5,6-DHET		↓	Zhang et al. (2017) [153]
	14,15-EET		↓	Zhang et al. (2017) [153]
	8-iso-PGF2α		↓	Zhang et al. (2017) [153]
	8-iso-PGE2		↓	Zhang et al. (2017) [153]
	2,3-dinor-8-iso-PGF2α		↑	Zhang et al. (2017) [153]
	GTA-446		↓	Ritchie et al. (2010) [168]
			↓	Hata et al. (2017) [170]
plasma	16:0	total lipids	↑	Okuno et al. (2013) [172]
		total lipids	↓	Butler et al. (2017) [87]
	18:0	total lipids	↓	Butler et al. (2017) [87]
	24:0	total lipids	↓	Okuno et al. (2013) [172]
	16:1 n-7	total lipids	↓	Butler et al. (2017) [87]
	18:1 n-9	total lipids	↓	Butler et al. (2017) [87]
	20:1	total lipids	↓	Okuno et al. (2013) [172]
	20:1	free fatty acids	↑	Shen et al. (2017) [89]
	18:2 n-6	total lipids	↓	Butler et al. (2017) [87]
	18:3 n-6	total lipids	↓	Butler et al. (2017) [87]
	20:2 n-6	free fatty acids	↑	Shen et al. (2017) [89]
	20:3 n-6	total lipids	↓	Butler et al. (2017) [87]
	20:5 n-3	total lipids	↓	Okuno et al. (2013) [172]
	22:4 n-6	free fatty acids	↑	Shen et al. (2017) [89]
	18:3 n-3	total lipids	↓	Butler et al. (2017) [87]
	Cer-d18:1/26:4		↑	Shen et al. (2017) [89]
	LPC-18:2		↓	Shen et al. (2017) [89]
	LPC-18:3		↓	Shen et al. (2017) [89]
			↓	Li et al. (2013) [178]
	PE-18:1/20:2		↓	Shen et al. (2017) [89]
	PE-18:2/18:1		↓	Shen et al. (2017) [89]
	PG-18:0/16:0		↑	Shen et al. (2017) [89]
	SM-38:8		↓	Shen et al. (2017) [89]
	SM-d18:1/24:1		↑	Shen et al. (2017) [89]
erythrocyte	18:0	total lipids	↑	Neoptolemos et al. (1988) [218]
	20:0	total lipids	↓	Okuno et al. (2013) [172]
	18:1 n-9	total lipids	↑	Neoptolemos et al. (1988) [218]
	24:1	total lipids	↑	Okuno et al. (2013) [172]
	20:4 n-6	total lipids	↓	Neoptolemos et al. (1988) [218]
	20:5 n-3	total lipids	↓	Okuno et al. (2013) [172]
adipose tissue	16:1 n-9	total lipids	↑	Cottet et al. (2015) [217]
	20:1	total lipids	↑	Neoptolemos et al. (1988) [218]
	18:3 n-6	total lipids	↑	Giuliani et al. (2014) [92]
	20:3 n-6	total lipids	↑	Giuliani et al. (2014) [92]
			↑	Cottet et al. (2015) [217]
	22:4 n-6	total lipids	↑	Giuliani et al. (2014) [92]
			↑	Okuno et al. (2013) [172]
	18:3 n-3	total lipids	↓	Giuliani et al. (2014) [92]
			↓	Cottet et al. (2015) [217]
	18:4 n-3	total lipids	↓	Giuliani et al. (2014) [92]
	22:5 n-3	total lipids	↑	Cottet et al. (2015) [217]

*Cer* ceramide, *DG* diacylglycerol, *DHET* dihydroxyeicosatrienoic acid, *DiHOME* dihydroxyoctadecenoic acid, *EET* epoxyeicosatrienoic acid, *GlcCer* glucosylceramide, *HETE* hydroxyeicosatetraenoic acid, *HODE* hydroxyoctadecadienoic acid, *HpETE* hydroperoxyeicosatetraenoic acid, *HpODE* hydroperoxyoctadecadienoic acid, *KODE* ketooctadecadienoic acid, *LPC* lysophosphatidylcholine, *LT* leukotriene, *LX* lipoxin, *PA* phosphatidic acid, *PC* phosphatidylcholine, *PE* phosphorylethanolamine, *PG* phosphatidylglycerol, *PGE/F* prostaglandin E/F, *PI* phosphatidylinositol, *PS* phosphatidylserine, *SM* sphingomyelin, *TG* triacylglycerol

## Abbreviations

AA: Arachidonic acid
ABCA1: ATP-binding cassette sub-family A 1
ACSL1: Long chain acyl-CoA synthetase 1
AGPAT1: 1-acyl-sn-glycerol-3-phosphate acyltransferase alpha
ALA: α-linolenic acid
AMPK: 5’AMP-activated protein kinase
APC: Adenomatous polyposis coli
ATGL: Adipose triacylglycerol lipase
Cer: Ceramide
CerS: Ceramidase synthase
CHα: Choline kinase α
CLA: Conjugated linoleic acid
CoA: Coenzyme A
COX: Cyclooxygenase
cPLA_2_: Cytosolic phospholipase A_2_
CRC: Colorectal cancer
CSC: Cancer stem cell
CYP450: Cytochrome p450
DGLA: Dihomo-γ-linolenic acid
DHA: Docosahexaenoic acid
EGFR: Epidermal growth factor receptor
ELOVL: Fatty acid elongase
EMT: Epithelial-mesenchymal transition
EPA: Eicosapentaenoic acid
FA: Fatty acid
FASN: Fatty acid synthase
FFA: Free fatty acid
FIT: Fecal immunochemical test
gFOBT: Guaiac-based fecal occult blood test
GLA: γ-linolenic acid
Glu: Glucose
HDL: High density lipoprotein
HETE: Hydroxyeicosatetraenoic acid
HNE: 4-hydroxynonenal
hPULCFA: Hydroxylated, polyunsaturated ultra-long-chain fatty acid
isoP: Isoprostane
IκBα: Kappa-light-chain-enhancer of activated B cells inhibitor
JNK1: c-Jun N-terminal protein kinase 1
LA: Linoleic acid
Lac: Lactose
LCFA: Long-chain fatty acid
LDL: Low density lipoprotein
LOX: Lipoxygenase
LPA: Lysophosphatidic acid
LPC: Lysophosphatidylcholine
LPP: Lipid peroxidation product
LT: Leukotriene
MCFA: Medium-chain fatty acid
MDA: Malondialdehyde
MLC2: Myosin regulatory light chain 2
mTOR: Mammalian target of rapamycin
MUFA: Monounsaturated fatty acid
NEFA: Non-esterified fatty acid
NF-κB: Kappa-light-chain-enhancer of activated B cells
OA: Oleic acid
OXPHO: Oxidative phosphorylation
PC: Phosphatidylcholine
PE: Phosphorylethanolamine
PG: Prostaglandin
PHL: Phospholipid
PKR: Protein kinase R
PL: Polar lipid
PPAR: Peroxisome proliferator-activated receptor
PUFA: Polyunsaturated fatty acid
RBC: Red blood cell
REIMS: Rapid evaporative ionization mass spectrometry
Rho/ROCK: Rho/Rho-associated coiled-coil containing protein kinase
ROC: Receiver operating characteristic
ROS: Reactive oxygen species
S1P: Sphingosine-1-phosphate
SAT: Subcutaneous adipose tissue
SCD: Stearoyl-CoA desaturase
SCFA: Short-chain fatty acid
SFA: Saturated fatty acid
SM: Sphingomyelin
SphK: Sphingosine kinase
SPL: Sphingolipid
SPM: Specialized pro-resolving mediator
TC: Total cholesterol
TG: Triacylglycerol
TLR: Toll-like receptor
VAT: Visceral adipose tissue
VLCFA: Very long-chain fatty acid
VLDL: Very-low-density-lipoprotein
YAMC: Young adult mouse colonic
